# Dynamic variations in COVID-19 with the SARS-CoV-2 Omicron variant in Kazakhstan and Pakistan

**DOI:** 10.1186/s40249-023-01072-5

**Published:** 2023-03-15

**Authors:** Qianqian Cui, Zhengli Shi, Duman Yimamaidi, Ben Hu, Zhuo Zhang, Muhammad Saqib, Ali Zohaib, Baikadamova Gulnara, Mukhanbetkaliyev Yersyn, Zengyun Hu, Shizhu Li

**Affiliations:** 1 School of Mathematics and Statistics, Ningxia University, Yinchuan, 750021 Ningxia China; 2 Chinese Academy of Sciences Key Laboratory of Special Pathogens and Biosafety, Wuhan Institute of Virology, Chinese Academy of Sciences, Wuhan, 430071 China; 3 State Key Laboratory of Desert and Oasis Ecology, Xinjiang Institute of Ecology and Geography, Chinese Academy of Sciences, Ürümqi, 830011 Xinjiang China; 4 Research Center for Ecology and Environment of Central Asia, Chinese Academy of Sciences, Ürümqi, 830011 Xinjiang China; 5 University of Chinese Academy of Sciences, Beijing, China; 6 College of Geography and Remote Sensing Sciences, Xinjiang University, Ürümqi, 830017 China; 7 Department of Clinical Medicine and Surgery, Faculty of Veterinary Science, University of Agriculture Faisalabad, Faisalabad, Pakistan; 8 Department of Microbiology, Faculty of Veterinary and Animal Sciences, The Islamia University of Bahawalpur, Bahawalpur, Pakistan; 9 Veterinary Medicine Department, Kazakh Agrotechnical University, Astana, Kazakhstan; 10 National Institute of Parasitic Diseases, Chinese Centre for Disease Control and Prevention (Chinese Centre for Tropical Diseases Research), NHC Key Laboratory of Parasite and Vector Biology, WHO Collaborating Centre for Tropical Diseases, National Centre for International Research On Tropical Diseases, Shanghai, 200025 China

**Keywords:** COVID-19, Pandemic, Omicron, Daily new confirmed cases, Cumulative confirmed cases, Simulation, Prediction

## Abstract

**Background:**

The ongoing coronavirus disease 2019 (COVID-19) pandemic caused by the severe acute respiratory syndrome-coronavirus 2 (SARS-CoV-2) and the Omicron variant presents a formidable challenge for control and prevention worldwide, especially for low- and middle-income countries (LMICs). Hence, taking Kazakhstan and Pakistan as examples, this study aims to explore COVID-19 transmission with the Omicron variant at different contact, quarantine and test rates.

**Methods:**

A disease dynamic model was applied, the population was segmented, and three time stages for Omicron transmission were established: the initial outbreak, a period of stabilization, and a second outbreak. The impact of population contact, quarantine and testing on the disease are analyzed in five scenarios to analysis their impacts on the disease. Four statistical metrics are employed to quantify the model’s performance, including the correlation coefficient (CC), normalized absolute error, normalized root mean square error and distance between indices of simulation and observation (DISO).

**Results:**

Our model has high performance in simulating COVID-19 transmission in Kazakhstan and Pakistan with high CC values greater than 0.9 and DISO values less than 0.5. Compared with the present measures (baseline), decreasing (increasing) the contact rates or increasing (decreasing) the quarantined rates can reduce (increase) the peak values of daily new cases and forward (delay) the peak value times (decreasing 842 and forward 2 days for Kazakhstan). The impact of the test rates on the disease are weak. When the start time of stage II is 6 days, the daily new cases are more than 8 and 5 times the rate for Kazakhstan and Pakistan, respectively (29,573 vs. 3259; 7398 vs. 1108). The impact of the start times of stage III on the disease are contradictory to those of stage II.

**Conclusions:**

For the two LMICs, Kazakhstan and Pakistan, stronger control and prevention measures can be more effective in combating COVID-19. Therefore, to reduce Omicron transmission, strict management of population movement should be employed. Moreover, the timely application of these strategies also plays a key role in disease control.

**Graphical abstract:**

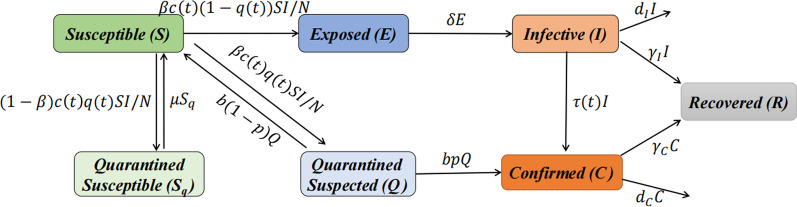

**Supplementary Information:**

The online version contains supplementary material available at 10.1186/s40249-023-01072-5.

## Background

The coronavirus disease 2019 (COVID-19) caused by severe acute respiratory syndrome coronavirus 2 (SARS-CoV-2) has rapidly spread worldwide over the past 3 years, with more than 623 million total confirmed cases and more than 6 million deaths (https://www.who.int/emergencies/diseases/novel-coronavirus-2019). As is well known, all viruses, including SARS-CoV-2, mutate over time, and the resulting variants may differ as to how easily the virus spreads and how severe the disease will be. In the end, SARS-CoV-2 variants may result in a decrease or loss of vaccine effectiveness and necessitate changes in public health and social policy measures [1–4].

The first four SARS-CoV-2 variants of concern were discovered in settings with high infection pressure before vaccines were available. The Alpha variant of concern (B.1.1.7) was detected in September 2020 in the United Kingdom, Beta (B.1.351) in May 2020 in South Africa, Gamma (P.1) in November 2020 in Brazil and Delta (B.1.617.2) in October 2020 in India [5] (https://www.who.int/en/activities/tracking-SARS-CoV-2-variants). On 26 November 2021, the World Health Organizations Technical Advisory Group on SARS-CoV-2 Virus Origin assigned Phylogenetic Assignment of Named Global Outbreak (PANGO) lineage B.1.1.529 as a variant of concern and gave it the Greek letter Omicron [6]. Rapid transmission of the SARS-CoV-2 Omicron variant has led to record-breaking incidence rates around the world and may portend large COVID-19 waves [4]. Since the first Omicron case was detected in Norway on 30 November 2021, it has been continuously observed in many countries, such as South Africa on 15 November 2021 [7, 8], the United States on 1 December 2021 and England in January 2022 [9, 10]. As of 20 January 2022, the Omicron variant had been discovered in 171 nations throughout the world, and it may spread faster than other variants due to its mutations [9].

In general, disease dynamic models constructed by ordinary differential equations or partial differential equations have a high ability to describe transmission features and forecast future changes. Therefore, nearly 3 years into the pandemic, exploring the transmission characteristics and predicting the future COVID-19 variants have received increasing attention with the use of mathematical models, such as disease dynamic models [11–15].

Disease dynamic models are essentially based on the disease transmission mechanism using mathematical equations (e.g., ordinary differential equations and partial differential equations), which have been widely employed to investigate how disease spreads [16–18]. In the less than 3 years of the COVID-19 pandemic, disease dynamic models have been applied to explore global and regional disease transmission features [11, 13, 15, 19].

Other studies have investigated the spread characteristics of COVID-19 using time series analysis models [20–22]. For example, with the advantages of COVID-19 data (e.g., hospital and vaccination data), Coccia [22] employed some simple and important statistical models to investigate the comparative analysis of the temporal dynamics and effects of the COVID-19 pandemic between 2020 and 2021 in Italy with different control measures. The results suggest that the COVID-19 pandemic is driven by seasonality and environmental factors that reduce negative effects in the summer, regardless of control measures and/or vaccination campaigns. Considering the microscopic social interactions among individuals, an exposure-risk-based model is developed to forecast the transmission trends of infectious respiratory diseases (e.g., COVID-19) [21].

In this study, we focus on the forecast variations of SARS-CoV-2 Omicron in two LMICs. Kazakhstan and Pakistan were chosen because they share borders with China and could cause problems for China if they are unable to prevent and control the disease. The widely used disease dynamic model constructed by ordinary differential equations will be employed to improve the accuracy of predicting future changes in COVID-19.

## Methods

### Study area and a brief analysis of COVID-19

Kazakhstan and Pakistan have close relationships with the Xinjiang Autonomous Region, China, in the Silk Belt and along the Silk Road. Kazakhstan has seven ports, including Ahertu Buick, Baktu Jeminay, Alashankou, Horgos, Dulata and Muzart. Pakistan has one, the Khunierab port. In 2020, the total population of Xinjiang, Kazakhstan and Pakistan was 25.89 million, 18.78 million and 220.89 million, respectively, according to the United Nations Statistics Division (data.un.org) and the National Bureau of Statistics of China (http://www.stats.gov.cn/).

Among the Central and South Asian countries, Kazakhstan and Pakistan play a great role in international trade, as shown in Additional file 1: Fig. S1. From 2000 to 2021, there were significant increases in the total import and export volumes of USD1.09 billion per year for China-Kazakhstan and $1.14 billion per year for China-Pakistan. In the 1st full year of the COVID-19 pandemic in 2020, the total import and export volume between China and Kazakhstan decreased by $494.54 million compared to 2019, and it decreased by $490.51 million for Pakistan. With the effective prevention and control of COVID-19, total imports, and exports for the two countries are trending up again.

In Kazakhstan, the first cases of COVID-19 were reported on March 13, 2020, and the first cases of the Omicron strain were detected on January 6, 2022. During the pandemic, the largest number of new cases in a day was 16,442 on January 21, 2022 (Additional file 1: Fig. S2a), and the cumulative confirmed cases total more than 1.4 million (Additional file 1: Fig. S2b). To prevent the spread of the disease, a state of emergency was declared and numerous nonpharmaceutical interventions (NPIs) (e.g., limiting public gatherings, physical distancing, lockdown, and quarantine) and universal mass vaccination was required. With dynamic control and prevention measurements in place, the number of COVID-19 cases have ebbed and flowed in multiple waves (Additional file 1: Fig. S2a).

In Pakistan, since the first case was reported on February 26, 2020, there have been at least five waves of COVID-19 with new cases peaking at more than 3000 a day. A rapid increase in COVID-19 was observed following the first reported case of the Omicron variant on December 9, 2021, a faster spread than the other four variants (i.e., Alpha, Beta, Gamma and Delta), which resulted in the largest number of cases in a day, 8183 on January 29, 2022 (Additional file 1: Fig. S3a). The cumulative confirmed cases stood at more than 1.5 million on October 14, 2022 (Additional file 1: Fig. S3b). A number of control and prevention strategies have been employed to control the spread of COVID-19 in the country, including NPIs and COVID-19 vaccination.

The Omicron variant is highly transmissible, as seen in both Kazakhstan and Pakistan (Additional file 1: Figs. S2a, 3a). To investigate Omicron transmission characteristics, we focused on the simulation and prediction of the stage of the variant’s spread for the two countries using the dynamic disease model. The study period was from January 6, 2022, to October 14, 2022. Because the NPIs in the two countries were determined by the variants features, the study period is divided into three stages: stage I from January 6 to March 17, stage II from March 18 to July 17 and stage III from July 18 to October 14.

### Dynamic disease model

**Fig. 1:**
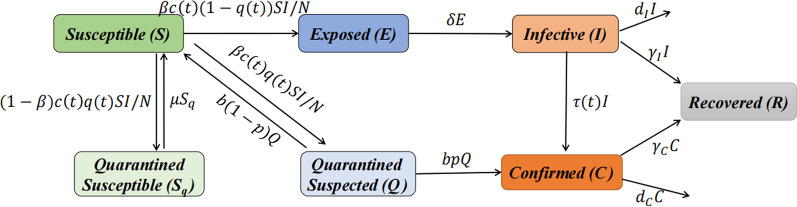
Flowchart of the COVID-19 dynamic model of Kazakhstan and Pakistan

Therefore, we also employ the dynamic disease model to simulate and predict the behavior of the COVID-19 Omicron variant in Kazakhstan and Pakistan. In constructing the model, according to the disease spread and the disease datasets collected in the two countries, the populations were divided into five groups, encompassing susceptible, exposed, infectious, confirmed and recovered individuals. Moreover, we considered three major factors that affected the COVID-19 variant’s behavior, including contact frequency, quarantine situation and disease testing requirements, which changed with time. The details of the model construction are provided in Fig. 1.

From the above analysis and the flowchart of COVID-19 transmission in the two countries, the disease dynamic model is constructed as follows.

1 $$\left\{\begin{array}{c}{S}^{^{\prime}}=-\beta c\left(t\right)\frac{S}{N}I-\left(1-\beta \right)c\left(t\right)q\left(t\right)\frac{S}{N}I+\mu {S}_{q}+b\left(1-p\right)Q, \\ {E}^{^{\prime}}=\beta c\left(t\right)\left(1-q\left(t\right)\right)\frac{S}{N}I-\delta E,\\ {I}^{^{\prime}}=\delta E-\tau \left(t\right)I-{\gamma }_{I}I-{d}_{I}I,\\ {{S}_{q}}^{^{\prime}}=\left(1-\beta \right)c\left(t\right)q\left(t\right)\frac{S}{N}I-\mu {S}_{q},\\ {Q}^{^{\prime}}=\beta c\left(t\right)q\left(t\right)\frac{S}{N}I-bQ,\\ {C}^{^{\prime}}=bpQ+\tau \left(t\right)I-{d}_{C}C-{\gamma }_{C}C,\\ {R}^{^{\prime}}={\gamma }_{I}I+{\gamma }_{C}C,\end{array}\right.$$

The time-dependent contact rate

$$c\left(t\right)=\left\{\begin{array}{c}\left({c}_{0}-{c}_{1}\right){e}^{-{r}_{1}^{c}t}+{c}_{1},\hspace{1em}0\le t<{t}_{0},\\ \left(c\left({t}_{0}\right)-{c}_{2}\right){e}^{-{r}_{2}^{c}\left(t-{t}_{0}\right)}+{c}_{2},\hspace{1em}{t}_{0}\le t<{t}_{1},\\ \left(c\left({t}_{1}\right)-{c}_{3}\right){e}^{-{r}_{3}^{c}\left(t-{t}_{1}\right)}+{c}_{3},\hspace{1em}t\ge {t}_{1},\end{array}\right.$$

time-dependent quarantined rate

$$q\left(t\right)=\left\{\begin{array}{c}\left({q}_{0}-{q}_{1}\right){e}^{-{r}_{1}^{q}t}+{q}_{1},\hspace{1em}0\le t<{t}_{0},\\ \left(q\left({t}_{0}\right)-{q}_{2}\right){e}^{-{r}_{2}^{q}\left(t-{t}_{0}\right)}+{q}_{2},\hspace{1em}{t}_{0}\le t<{t}_{1},\\ \left(q\left({t}_{1}\right)-{q}_{3}\right){e}^{-{r}_{3}^{q}\left(t-{t}_{1}\right)}+{q}_{3},\hspace{1em}t\ge {t}_{1},\end{array}\right.$$

time-dependent detection rate

$$\frac{1}{\tau \left(t\right)}=\left\{\begin{array}{c}\left(\frac{1}{{\tau }_{0}}-\frac{1}{{\tau }_{1}}\right){e}^{-{r}_{1}^{\tau }t}+\frac{1}{{\tau }_{1}},\hspace{1em}0\le t<{t}_{0},\\ \left(\frac{1}{\tau \left({t}_{0}\right)}-\frac{1}{{\tau }_{2}}\right){e}^{-{r}_{2}^{\tau }\left(t-{t}_{0}\right)}+\frac{1}{{\tau }_{2}},\hspace{1em}{t}_{0}\le t<{t}_{1},\\ \left(\frac{1}{\tau \left({t}_{1}\right)}-\frac{1}{{\tau }_{3}}\right){e}^{-{r}_{3}^{\tau }\left(t-{t}_{1}\right)}+\frac{1}{{\tau }_{3}}\hspace{1em}t\ge {t}_{1}.\end{array}\right.$$

To quantify the model’s simulation and prediction performance, the correlation coefficient (CC), absolute error (AE) and root mean square error (RMSE) are employed. The overall performance is evaluated by the distance between indices of simulation and observation (DISO), which is based on the Euclidean distance and flexible determination of statistical metrics and their numbers from the Da Dao Zhi Jian concept [24–26]. The DISO equation is provided as follows.

$$DISO=\sqrt{{\left(CC-1\right)}^{2}+NA{E}^{2}+NRMS{E}^{2}}$$

where NAE and NRMSE are normalized by the averaged values of the observed time series.

## Results

In this section, we first simulate and predict the COVID-19 variations in both countries using model (3.1). Then, the scenario analysis results of different contact rates, quarantine rates and test rates in four scenarios are provided, including the scenarios about decreasing (or increasing) the NPIs when compared with the baseline (the present situation). Moreover, we only adjusted the start time points (i.e., *t*_0_ and *t*_1_) of stage II and stage III and kept the same NPIs and vaccines as the present situation to illustrate the impact of NPIs and vaccines employed at different time points on disease transmission. All the analyses focus on the daily new confirmed cases and cumulative confirmed cases. The following are the specific results (Table 1).

**Table 1:** Parameter estimates for the COVID-19 epidemic in Kazakhstan and Pakistan

Parameter	Definitions	Estimated values		Source
		Kazakhstan	Pakistan	
*β*	Probability of transmission per contact	0.089	0.0713	Estimated
*µ*	Release rate of quarantined uninfected contact	1/14	1/14	[19]
*c*_0_	Contact rate at the initial time	20.3	23.9	Estimate
*c*_1_(*c*_3_)	Minimum contact rate in stage I (III)	6.8(19.1)	12(9)	Estimated
*c*_2_	Maximum contact rate in stage II	16.5	18.3	Estimated
$r_{1}^{c} \left( {r_{2}^{c} ,r_{3}^{c} } \right)$	Exponential rate of contact rate under stage I (II, III)	0.01(0.02, 0.2)	0.08(0.04,0.25)	Estimated
*q*_0_	Quarantined rate of exposed individuals at the initial time	0.009	0.005	Estimated
*q*_1_(*q*_3_)	Maximum quarantined rate of exposed individuals in stage I (III)	0.8(0.62)	0.5(0.6)	Estimated
*q*_2_	Minimum quarantined rate of exposed individuals in stage II	0.2	0.25	Estimated
$r_{1}^{q} \left( {r_{2}^{q} ,r_{3}^{q} } \right)$	Exponential rate of quarantined rate of exposed individuals under stage I (II, III)	0.08(0.01,0.04)	0.05(0.04,0.1)	Estimated
b	Detection rate of the quarantined suspected class	0.2	0.2	Estimated
*P*	Transition rate of quarantined suspected class to the confirmed class	0.9	0.9	Estimated
*δ*	Transition rate of exposed individuals to the infected class	1/3.42	1/3.42	[23]
*τ*_0_	Initial diagnosis rate at initial time	0.0214	0.07	Estimated
*τ*_1_(*τ*_3_)	Fastest diagnosis rate in stage I (III)	0.8(0.43)	0.6(0.3)	Estimated
*τ*_2_	Fastest diagnosis rate in stage II	0.1	0.12	Estimated
$r_{1}^{\tau } \left( {r_{2}^{\tau } ,r_{3}^{\tau } } \right)$	Exponential rate of diagnosis rate in stage I (II, III)	0.2(0.1,0.1)	0.2(0.09,0.001)	Estimated
*γ*_*I*_	Recovery rate of infected individuals	0.28	0.228	Estimated
*d*_*I*_	Disease-induced death rate of infected individuals	0.00002	0.00005	Estimated
*γ*_*C*_	Recovery rate of confirmed individuals	0.08	0.06	Estimated
*d*_*C*_	Disease-induced death rate of confirmed individuals	0.000145	0.000366	Estimated
*t*_0_(*t*_1_)	The starting time of stage II(III)	71(193)	113(170)	Data

Initial values	Definitions	Estimated value		Source
		Kazakhstan	Pakistan	
*N* (0)	Initial total population	1*.*9 × 10^7^	2*.*21 × 10^8^	Data
*S*(0)	Initial susceptible population	1*.*792 × 10^7^	2*.*197 × 10^8^	Estimated
*E*(0)	Initial exposed population	2750	1950	Estimated
*I*(0)	Initial infected population	1220	1120	Estimated
*S*_*q*_(0)	Initial quarantined susceptible population	4976	10,707	Estimated
*Q*(0)	Initial quarantined suspected population	420	180	Estimated
*C*(0)	Initial confirmed population	94,945	6521	Data
*R*(*o*)	Initial recovered population	961,806	1*.*263 × 10^6^	Data

The prime (,) denotes the differentiation with respect to time *t*

*S* susceptible; *S*_*q*_ quarantined susceptible; *E* exposed; *Q* quarantined suspected; *I* infected; *C* confirmed; *R* recovered

### Simulation and prediction analysis of COVID-19

**Fig. 2:**
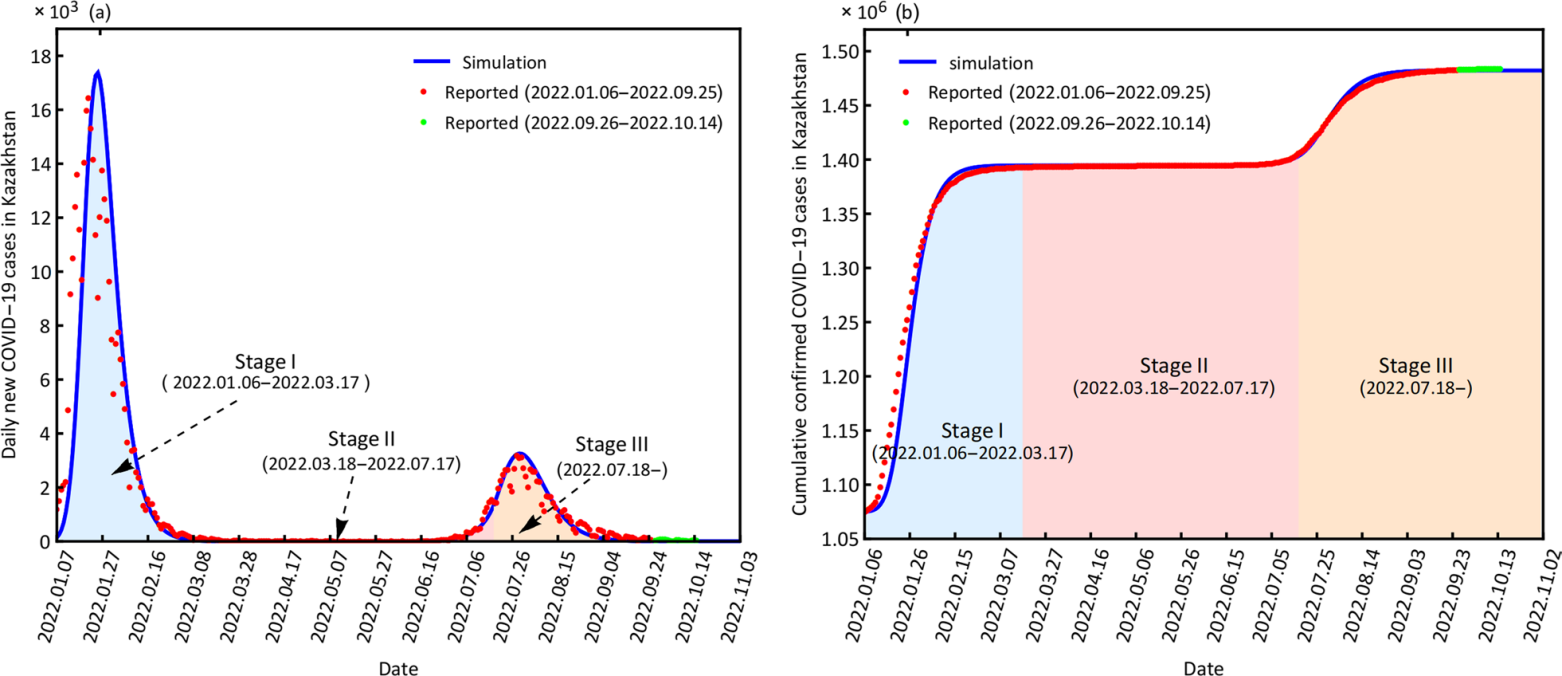
Simulation and prediction of daily new confirmed cases (**a**) and cumulative confirmed cases (**b**) Kazakhstan

**Fig. 3:**
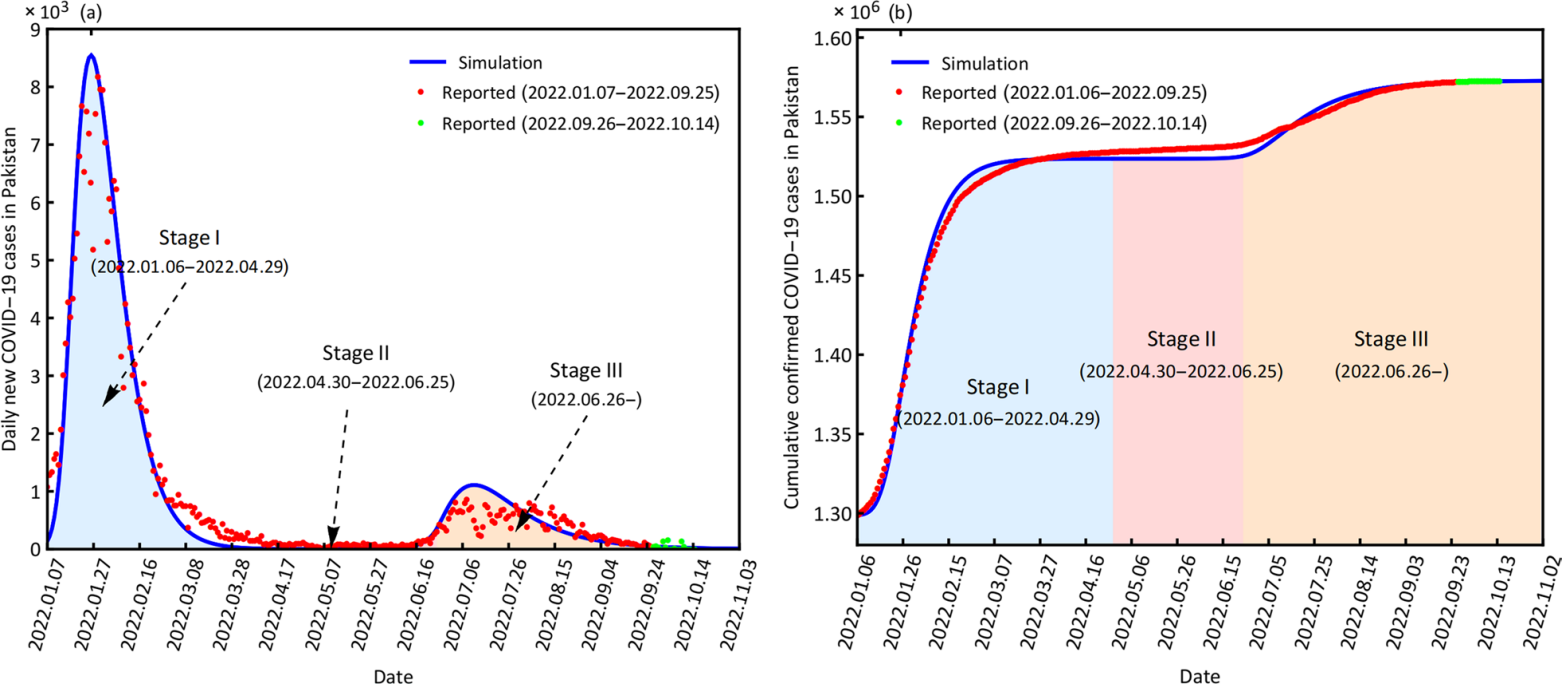
Simulation and prediction of daily new confirmed cases (**a**) and cumulative confirmed cases (**b**) for Pakistan

The study period includes the simulation period from January 6, 2022, to September 25, 2022, and the prediction period from September 26, 2022, to October 14, 2022. The simulation and prediction results for the two countries are displayed in Figs. 2 and 3.

Our model (3.1) captures the historical COVID-19 transmissions in the two countries well (Figs. 2, 3). The CC values are larger than 0.90 for cumulative confirmed cases. Most NAE values are zero, NRMSE values are smaller than 2 and DISO values are smaller than 0.5, which indicates that the dynamic disease model (3.1) has a very comprehensive performance in simulating and predicting the daily new confirmed cases and cumulative confirmed cases (Table 2).

**Table 2:** Evaluation results of the simulation and prediction of daily new confirmed cases and cumulative confirmed cases for Kazakhstan and Pakistan

Country	Case	Time period	CC	NAE	NRMSE	DISO
Kazakhstan	Daily new	01.06–09.25	0.92	0.00	0.91	0.43
		09.26–10.14	0.35	-0.97	1.04	0.49
	Cumulative	01.06–09.25	0.99	0.00	0.01	0.33
		09.26–10.14	0.98	0.00	0.00	0.33
Pakistan	Daily new	01.06–09.25	0.98	0.00	0.42	0.36
		09.26–10.14	0.32	-0.14	1.39	0.48
	Cumulative	01.06–09.25	1.00	0.00	0.00	0.33
		09.26–10.14	0.97	0.00	0.00	0.32

*CC* correlation coefficient; *DISO* distance between indices of simulation and observation; *AE* absolute error; *RMSE* rote mean square error

### Scenario analysis of COVID-19

#### COVID-19 variations at different contact rates

**Fig. 4:**
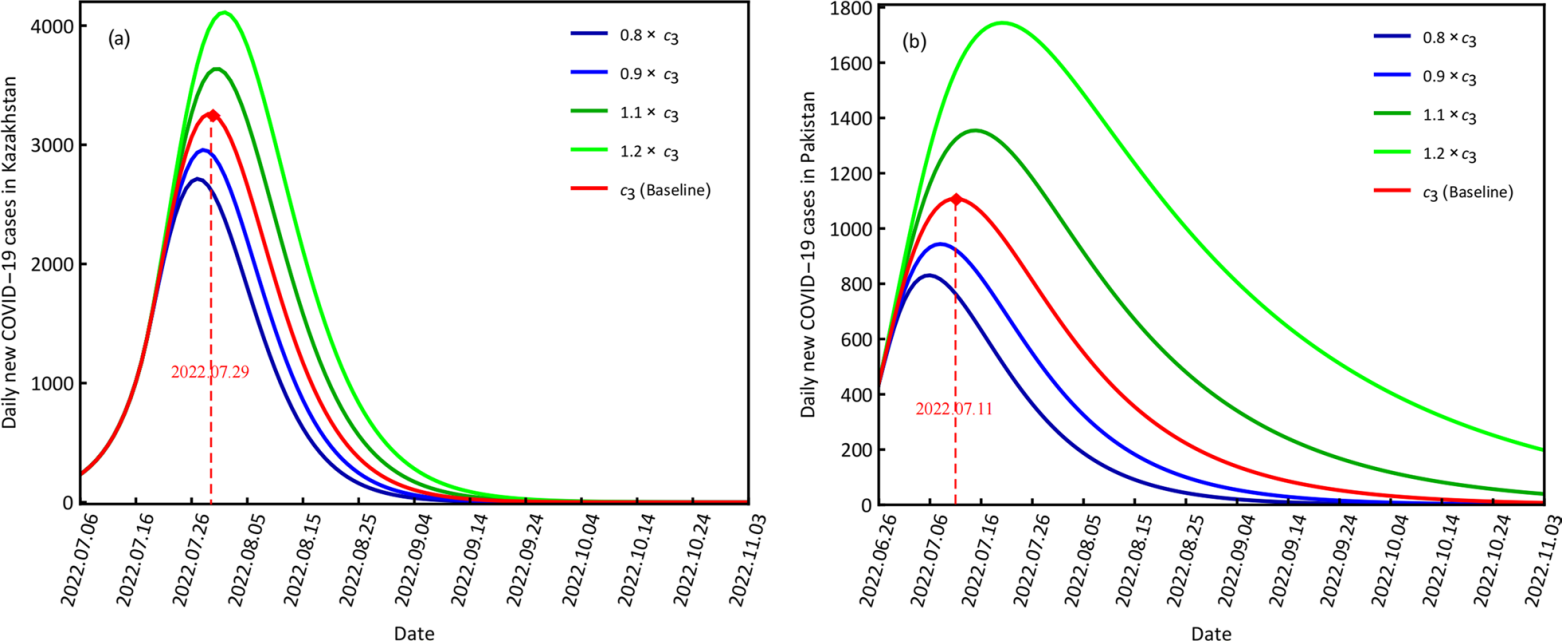
Scenario results of the different contact rates for daily new COVID-19 cases in Kazakhstan (**a**) and Pakistan (**b**)

To investigate the impact of the different contact rates on COVID-19 transmission from the perspective of daily new confirmed cases, we set four scenarios: 0.8*c*_3_, 0.9*c*_3_, 1.1*c*_3_ and 1.2*c*_3_, which were used in comparisons with the baseline (*c*_3_).The smaller contact rates are shown to reduce the peak values of the daily new confirmed cases and increase the corresponding time points, and larger contract rates resulted in larger peak values and delayed corresponding time points for both countries (Fig. 4). For example, when the contact rate was decreased to 0.8*c*_3_, the peak values were 2714 for Kazakhstan and 831 for Pakistan with the corresponding time points of July 27, 2022 and July 6, 2022; when the contact rate was increased to 1.2*c*_3_, the peak values were 4111 for Kazakhstan and 1744 for Pakistan with the corresponding time points of August 1, 2022 and July 20, 2022 (Fig. 4, Table 3). For baseline *c*_3_, the peak values of Kazakhstan and Pakistan are 3259 and 1108, respectively, with corresponding time points of July 29, 2022, and July 11, 2022.

**Table 3:** Scenario results of the different contact rates c3 for Kazakhstan and Pakistan, where peak value is the daily new confirmed cases

Country	Scenarios	Peak value	Time point
Kazakhstan	0.8*c*_3_	2417	2022.07.27
	0.9*c*_3_	2957	2022.07.28
	*c*_3_	3259	2022.07.29
	1*.*1*c*_3_	3635	2022.07.31
	1*.*2*c*_3_	4111	2022.08.01
Pakistan	0.8*c*_3_	831	2022.07.06
	0.9*c*_3_	944	2022.07.08
	*c*_3_	1108	2022.07.11
	1*.*1*c*_3_	1355	2022.07.15
	1*.*2*c*_3_	1744	2022.07.20

#### COVID-19 variations at different quarantine rates

**Fig. 5:**
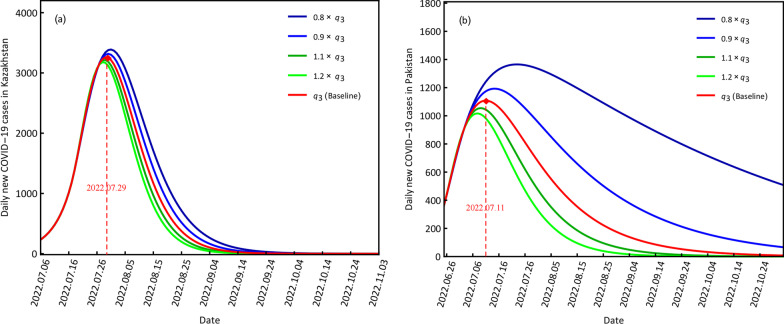
Scenario results of the different quarantine rates for daily new COVID-19 cases in Kazakhstan (**a**) and Pakistan (**b**)

For the different quarantine rates, the four scenario values were 0*.*8*q*_3_, 0.9*q*_3_, 1.1*q*_3_ and 1.2*q*_3_, and the baseline was (*q*_3_). The smaller quarantine rates in controlling disease transmission suggest that the peak value of daily new confirmed cases will become larger and the corresponding time points will be delayed compared with the baseline condition (Fig. 5). In contrast, stronger quarantine measures meant smaller peak values and forward time points for both countries. In particular, when the quarantine rate was 0*.*8*q*_3_, the peak values were 3389 for Kazakhstan and 1365 for Pakistan, with corresponding time points of July 31, 2022, and July 22, 2022; when the quarantine rate was 1.2*q*_3_, the peak values were 3197 for Kazakhstan and 1017 for Pakistan, with corresponding time points of July 28, 2022, and July 8, 2022 (Fig. 5, Table 4). For the baseline *q*_3_, the peak values of Kazakhstan and Pakistan were 3259 and 1108, respectively, with corresponding time points of July 29, 2022, and July 11, 2022.

**Table 4:** Scenario results of the different quarantine rates *q*_3_ for Kazakhstan and Pakistan

Country	Scenario	Peak value	Time
Kazakhstan	0.8*q*_3_	3389	2022.07.31
	0.9*q*_3_	3317	2022.07.30
	*q*_3_	3259	2022.07.29
	1*.*1*q*_3_	3215	2022.07.29
	1*.*2*q*_3_	3179	2022.07.28
Pakistan	0.8*q*_3_	1365	2022.07.22
	0.9*q*_3_	1193	2022.07.14
	*q*_3_	1108	2022.07.11
	1*.*1*q*_3_	1055	2022.07.09
	1*.*2*q*_3_	1017	2022.07.08

#### COVID-19 variations at different detection rates

**Fig. 6:**
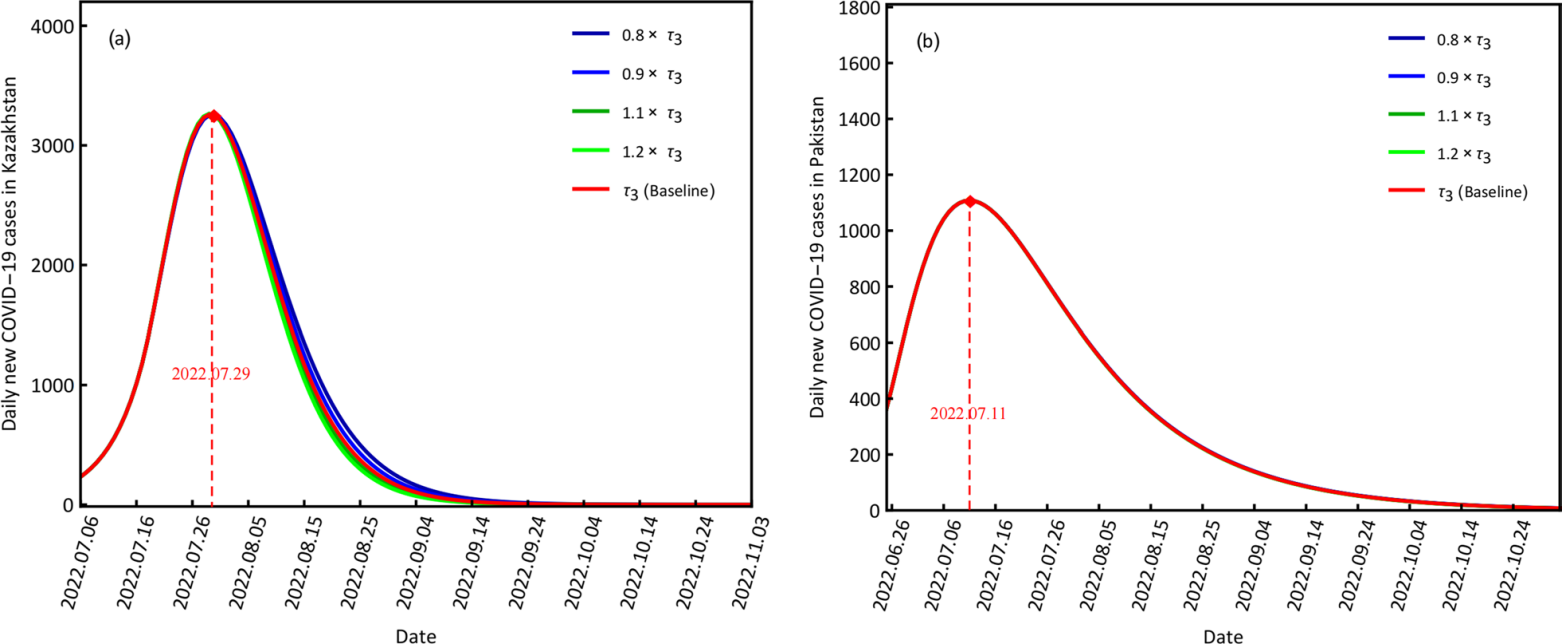
Scenario results of the different detection rates for daily new COVID-19 cases in Kazakhstan (**a**) and Pakistan (**b**)

The impact of detection rates on disease transmission are provided in Fig. 6 with four scenarios of 0*.*8*τ*_3_, 0*.*9*τ*_3_, 1*.*1*τ*_3_ and 1*.*2*τ*_3_. Small detection rates indicate weak disease screening ability, and large detection rates suggest strong disease screening ability.

Small differences existed after changing the detection rates for the daily new confirmed cases in both countries, which may have been caused by their weak primary detection abilities. At the baseline *τ*_3_, the peak values and the corresponding time points of Kazakhstan and Pakistan were the same as the baselines of *c*_3_ and *q*_3_.

#### COVID-19 variations at different start times of stage II

**Fig. 7:**
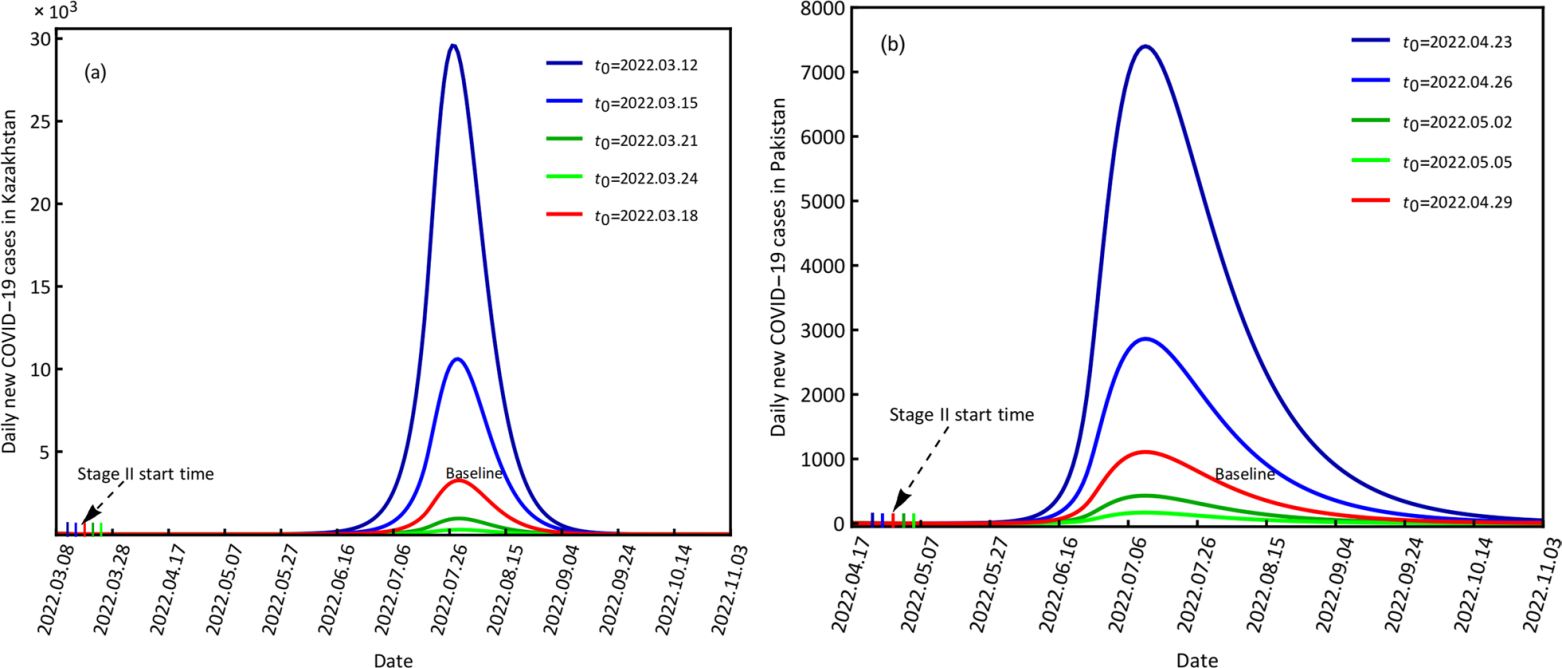
Scenario results of the different start times of stage II for daily new COVID-19 cases in Kazakhstan (**a**) and Pakistan (**b**)

Keeping the same control and prevention measures, we only changed the start time for different stages with a time interval of 3 days because of the incubation period of the Omicron variant. The start times for stage II were set as March 12, March 15, March 21, and March 24 and compared with the baseline of March 18 for Kazakhstan; April 23, April 26, May 2 and May 5 were compared with the baseline of April 29 for Pakistan (Fig. 7). Combined with the contact rates and quarantine rates at stage II, when the start time was brought forward, it had the forward maximum contact rate and the forward minimum quarantine rate, which would cause more infection than the baseline condition. When the start time was delayed, it had a delayed maximum contact rate and a delayed minimum quarantine rate, which would cause fewer infections. For example, when the start times were March 12 and April 23 for Kazakhstan and Pakistan, the peak values of daily new confirmed cases were 29,573 and 7398 for the two countries, which were more than 8 and 5 times those at the baselines. When the start times were March 24 and May 5 for Kazakhstan and Pakistan, the peak values of daily new confirmed cases were 277 and 168 for the two countries, respectively, which were fewer than those at the baselines (Fig. 7, Table 5).

**Table 5:** Scenario results of the different start times *t*_0_ for Kazakhstan and Pakistan, where *t*_0_ − n means before n days of *t*_0_, and *t*_0_ + n means after n days of *t*_0_, n = 3, 6

Country	Scenario	Peak value	Time point
Kazakhstan	*t*_0_ − 6	29,573	2022.07.27
	*t*_0_ − 3	10,608	2022.07.29
	*t*_0_	3259	2022.07.29
	*t*_0_ + 3	954	2022.07.29
	*t*_0_ − 6	277	2022.07.29
Pakistan	*t*_0_ − 6	7398	2022.07.11
	*t*_0_ − 3	2862	2022.07.11
	*t*_0_	1108	2022.07.11
	*t*_0_ + 3	431	2022.07.11
	*t*_0_ + 6	168	2022.07.11

#### COVID-19 variations at different start times of stage III

**Fig. 8:**
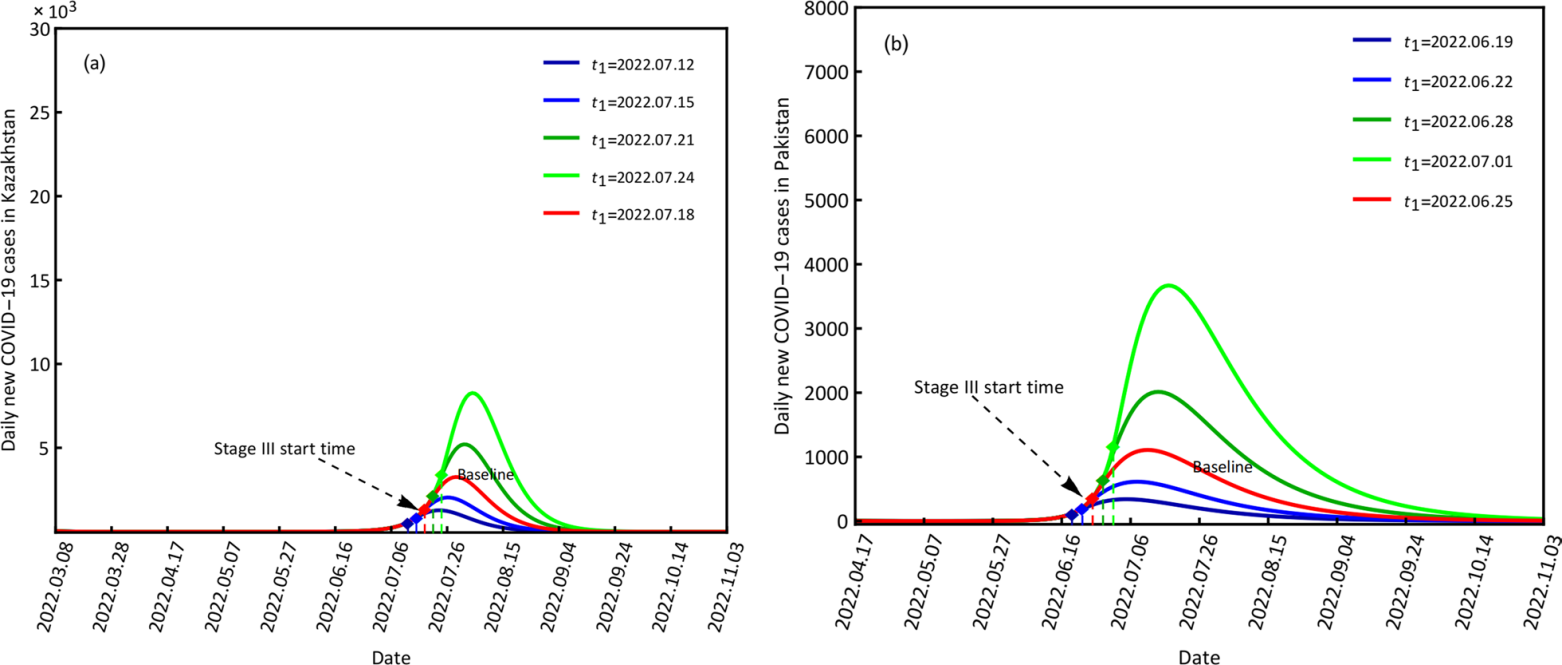
Scenario results of the different start times of stage III for daily new COVID-19 cases in Kazakhstan (**a**) and Pakistan (**b**)

The start times of stage III were set as July 12, July 15, July 21, and July 24 compared with the baseline of July 18 for Kazakhstan and June 19, June 22, June 28 and June 1 compared with the baseline of June 25 for Pakistan (Fig. 8). Combined with the contact rates and quarantined rates at stage II, when the start time was brought forward, it had the forward minimum contact rate and the forward maximum quarantined rate, which would cause fewer infections than the baseline condition. When the start time was delayed, it had a delayed minimum contact rate and a delayed maximum quarantine rate, which would cause more infection. For example, when the start times were July 12 and June 19 for Kazakhstan and Pakistan, the peak values of daily new confirmed cases were 1276 and 341 for the two countries, respectively, which were fewer than those at the baselines. When the start times were July 24 and July 1 for Kazakhstan and Pakistan, the peak values of daily new confirmed cases were 8267 and 3670 for the two countries with time points of August 4, 2022, and July 17, 2022, respectively, which were more than those at the base lines and the delayed time points (Fig. 8, Table 6)

**Table 6:** Scenario results of the different start times *t*_1_ for Kazakhstan and Pakistan

Country	Scenario	Peak value	Time point
Kazakhstan	*t*_1_ − 6	1276	2022.07.23
	*t*_1_ − 3	2037	2022.07.26
	*t*_1_	3259	2022.07.29
	*t*_1_ + 3	5207	2022.08.01
	*t*_1_ − 6	8267	2022.08.04
Pakistan	*t*_1_ − 6	341	2022.07.22
	*t*_1_ − 3	613	2022.07.14
	*t*_1_	1108	2022.07.11
	*t*_1_ + 3	2013	2022.07.09
	*t*_1_ + 6	3670	2022.07.08

## Discussion

The ongoing COVID-19 pandemic is still having a great impact on lives and livelihoods worldwide, and its control and prevention face huge challenges because SARS-CoV-2 will continue to evolve and attempt to evade immunity. Each new variant has demonstrated this in waves. Therefore, timely, accurate, and comprehensive estimates of the daily new confirmed cases and cumulative confirmed cases are essential for understanding the determinants of past infection, current transmission patterns, and future infection variations. For the control and prevention of COVID-19, NPIs rely on reducing contact between infected and susceptible individuals through mass social distancing, including restrictions on social gatherings, stay-at-home orders, lockdowns, closures of schools and business, travel restrictions, increased testing, active monitoring, contact tracing, and other isolation measures [27–31]. NPIs, especially lockdowns, can effectively reduce the reproduction of COVID-19 cases [32–35]. For the NPIs of Kazakhstan and Pakistan, we chose the contact rate, quarantine rate, and test rate to explore their impact on disease transmission. The results suggest that reducing the contact rates or increasing the quarantine rates can largely decrease the daily new confirmed cases, which is consistent with previous studies [29, 30, 32]. However, an increase in the test rate has a weak impact on disease transmission, which may be caused by the strong quarantine measures.

Almost 3 years into the pandemic, several COVID-19 vaccines have received emergency use listing or authorization by regulatory authorities and the World Health Organization based on the vaccine efficacy results from randomized controlled trials [36–39]. There is a realistic expectation that the global effort in vaccination will bring the pandemic caused by SARS-CoV-2 under control [15, 40]. COVID-19 vaccines combined with NPIs are very effective in reducing disease transmission, the risks of severe disease and mortality from the Omicron variant [6, 41, 42]. For Kazakhstan and Pakistan, the vaccination rates are mainly dependent on their low developed economies, which are lower than those of highly developed countries. Moreover, it is very difficult to obtain specific vaccination data about the two countries. Therefore, we will explore the impact of COVID-19 vaccines on disease transmission when data are available in the future.

For any disease, prevention plays a very important role in protecting human health, which demands the establishment of a new disease warning system with comprehensive early warning information about diseases. In recent years, health concepts composed of the environment and human and wild animal health have been proposed and widely developed in the control and prevention of human diseases, especially zoonoses [23, 43, 44]. Compared with the traditional disease warning system, the “one health model” claims to detect and monitor early warning information related to diseases, such as environmental factors (e.g., land use and land cover, temperature, precipitation and wind) and wild animal factors (e.g., wild animal population, density and behaviors) [45, 46]. COVID-19 is also a zoonosis that may correlate with climate factors and wild animals [45, 47]. Hence, the one-health COVID-19 model with climate factors and wild animal factors should be considered to provide more early warning information and reduce disease transmission.

For the forecasting of COVID-19 transmissions, in addition to the widely used dynamic models using ordinary differential equations, there are some other models considering the factor impacts on the transmission mechanism [21, 48, 49]. For example, an exposure risk-based model was established to explore COVID-19 transmission characteristics [21]. A moving averages model (MM7) has been used to detect the health policy of full lockdowns and a vast campaign of vaccinations [22]. However, these studies generally need more datasets to better explain the disease transmission mechanism, such as vaccination data and hospitalization data [22].

Our results indicate that model (2.1) has a good ability to capture the COVID-19 variations in the two countries with high CC values and very small DISO values. However, there are still some differences between our predicted data and the real-world data from September 26, 2022, to October 14, 2022. For example, prediction of the daily new data is different from the real-world data with low CC values, which may be caused by the short time period compared to the more than nine months in the simulated period (Table 2). If more datasets about COVID-19 in Kazakhstan and Pakistan become available, a comprehensive analysis will be provided in our future study. Now, we have to use the limited data to investigate COVID-19 transmission and predict the future tendencies using the dynamic model.

Some limitations exist in our study. For example, model (2.1) can be improved from the aspects of social distance, vaccination and mask use in the two countries. Moreover, some important parameters are estimated in our study, such as the contact rate and recovery rate of confirmed cases of COVID-19. More information about these aspects of disease transmission in the two countries is needed and can help establish a more accurate model.

## Conclusions

In this study, the dynamic variations in COVID-19 transmission with the omicron variant in Kazakhstan and Pakistan are explored with a dynamic disease model (2.1) derived by differential equations. First, the simulation and prediction of the disease are analyzed. Then, the impact of contact, quarantine and test rates are analyzed based on five different scenarios. Moreover, we explore the scenario results of COVID-19 variations with the control and prediction measurements at different time points. The main results are concluded as follows.

1. The dynamic model established by ordinary differential equations has high performance in simulating and predicting COVID-19 transmission in the two countries, including multiple wave variations. The simulation CC values of the daily new confirmed cases and the cumulative confirmed cases are higher than 0.9, and the DISO values are smaller than 0.5.
2. According to the scenario analysis of different contact rates and quarantine rates, disease transmission can be reduced by decreasing the contact rates and increasing the quarantine rates. In fact, the increased contact rates indicate decreased quarantine rates. The reduced contact rates largely indicate stronger quarantine measures.
3. Moreover, with these same magnitudes, the timely application of control and prevention strategies plays a key role in disease transmission based on the different start time points of Stage II and Stage III.

To fight against the COVID-19 pandemic, vaccines have been extensively deployed across most large countries in the world. Constrained by the vaccine datasets of Kazakhstan and Pakistan, the impact of the vaccine is not considered in our model. In the future, population movements and vaccines will be included in our model if these data become available.

## Supplementary Information

**Additional** **file 1.** Supplemenatry figures.

## Data Availability

The data will be available upon requested to first author.

## Abbreviations

COVID-19: Coronavirus disease 2019
SARS-CoV-2: Syndrome-coronavirus 2
CC: Correlation coefficient
DISO: Distance between indices of simulation and observation
PANGO: Phylogenetic Assignment of Named Global Outbreak
AE: Absolute error
RMSE: Rote mean square error
NPIs: Non-pharmaceutical interventions
