# Supplementary material for: Dynamic variations in COVID-19 with the SARS-CoV-2 Omicron variant in Kazakhstan and Pakistan
Source: Infect Dis Poverty. 2023 Mar 15;12:18. doi: 10.1186/s40249-023-01072-5 (PMC10014408; doi:10.1186/s40249-023-01072-5)
Supplement: Supplementary file 1 — Additional file 1. Supplemenatry figures. [file 40249_2023_1072_MOESM1_ESM.docx]

Additional Information for

Dynamic variations in COVID-19 with the SARS-CoV-2 Omicron Variant in Kazakhstan and Pakistan

Qianqian Cui^1^, Zhengli Shi^2^, Duman Yimamaidi^3^*^,^*^4^*^,^*^5^, Ben Hu^2^, Zhuo Zhang^6^, Muhammad Saqib^7^, Ali Zohaib^8^, Baikadamova Gulnara^9^, Mukhanbetkaliyev Yersyn^9^, Zengyun Hu^3^*^,^*^4^*^,^*^5^*^∗^*，Shizhu Li^10*^

1School of Mathematics and Statistics, Ningxia University, Yinchuan, Ningxia 750021, China

2 Chinese Academy of Sciences Key Laboratory of Special Pathogens and Biosafety, Wuhan Institute of Virology, Chinese Academy of Sciences, Wuhan, 430071

3 State Key Laboratory of desert and Oasis Ecology, Xinjiang Institute of Ecology and Geography, Chinese Academy of Sciences, Urumqi, Xinjiang 830011, China

4Research Center for Ecology and Environment of Central Asia,

Chinese Academy of Sciences, Urumqi, Xinjiang 830011, China

5University of Chinese Academy of Sciences, Beijing, China

6College of Geography and Remote Sensing Sciences, Xinjiang University, Urumqi 830017, China 7Department of Clinical Medicine and Surgery, Faculty of Veterinary Science, University of Agriculture Faisalabad

8Department of Microbiology, Faculty of Veterinary and Animal Sciences, The Islamia University of Bahawalpur

9Veterinary Medicine Department, Kazakh Agrotechnical University, Astana, Kazakhstan

10National Institute of Parasitic Diseases, Chinese Centre for Disease Control and Prevention

(Chinese Centre for Tropical Diseases Research), NHC Key Laboratory of Parasite and Vector Biology, WHO Collaborating Centre for Tropical Diseases,

National Centre for International Research on Tropical Diseases, Shanghai 200025, China

*Corresponding author: Zengyun Hu, Email: [huzengyun@ms.xjb.ac.cn](mailto:huzengyun@ms.xjb.ac.cn); Shizhu Li, Email: lisz@chinacdc.cn

**Additional Figure (Figure S) Captions**

Figure S1: The total import and export volume of China-Kazakhstan and China-Pakistan during the period of 2000-2021. The data is from the National Bureau of [Statistics(http://stats.go](http://stats.gov.cn/))v.cn/)

Figure S2: COVID-19 variations of Kazakhstan from March 13, 2020 to October 14, 2022, (a) for daily new cases and (b) for cumulated cases.

Figure S3: COVID-19 variations of Pakistan from February 26, 2020 to October 14, 2022, (a) for daily new cases and (b) for cumulated cases.


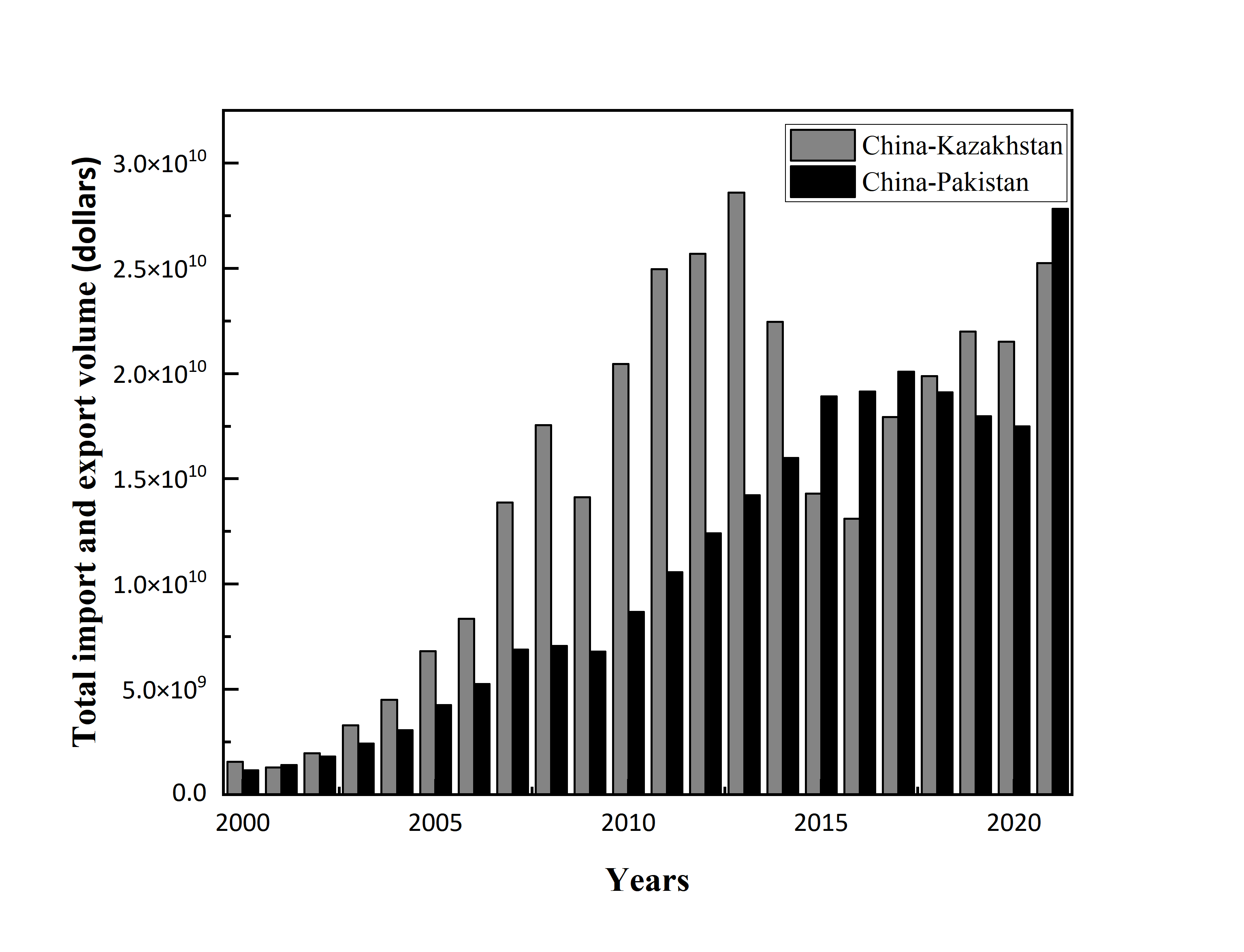


Figure S1: The total import and export volume of China-Kazakhstan and China-Pakistan during the period of 2000-2021. The data is from the National Bureau of [Statistics(http://stats.go](http://stats.gov.cn/))v.cn/)


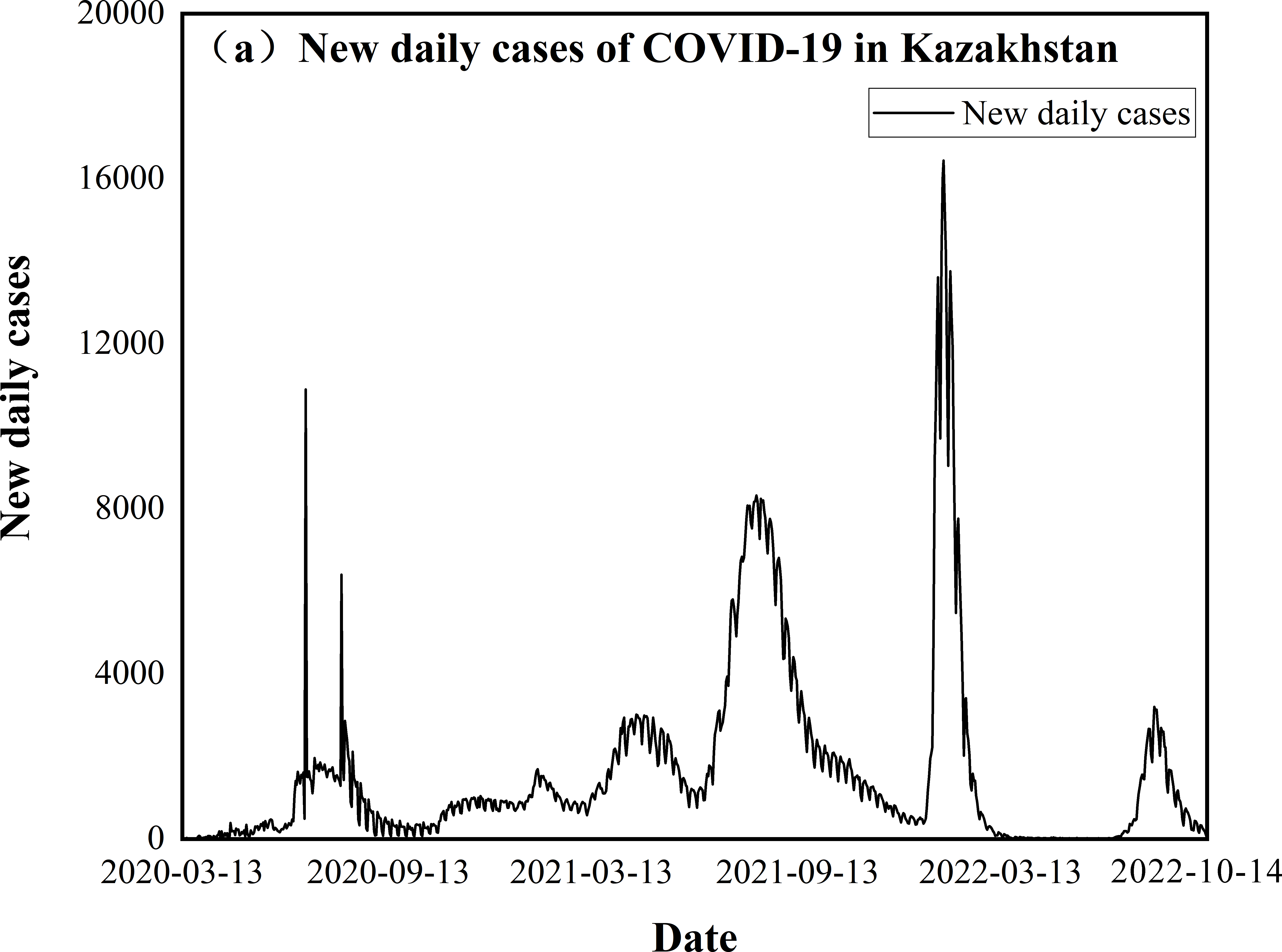

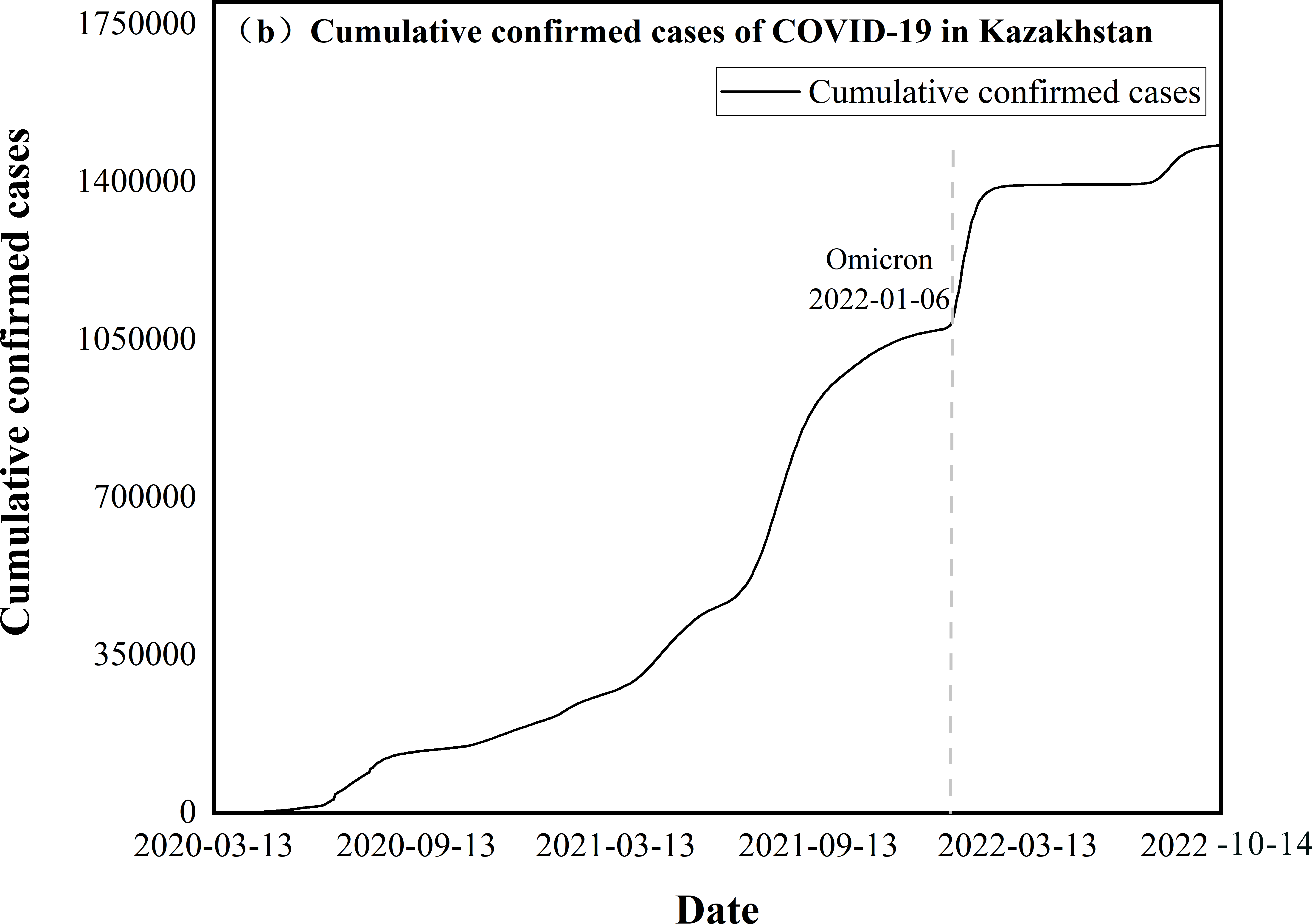


Figure S2: COVID-19 variations of Kazakhstan from March 13, 2020 to October 14, 2022, (a) for daily new cases and (b) for cumulated cases.


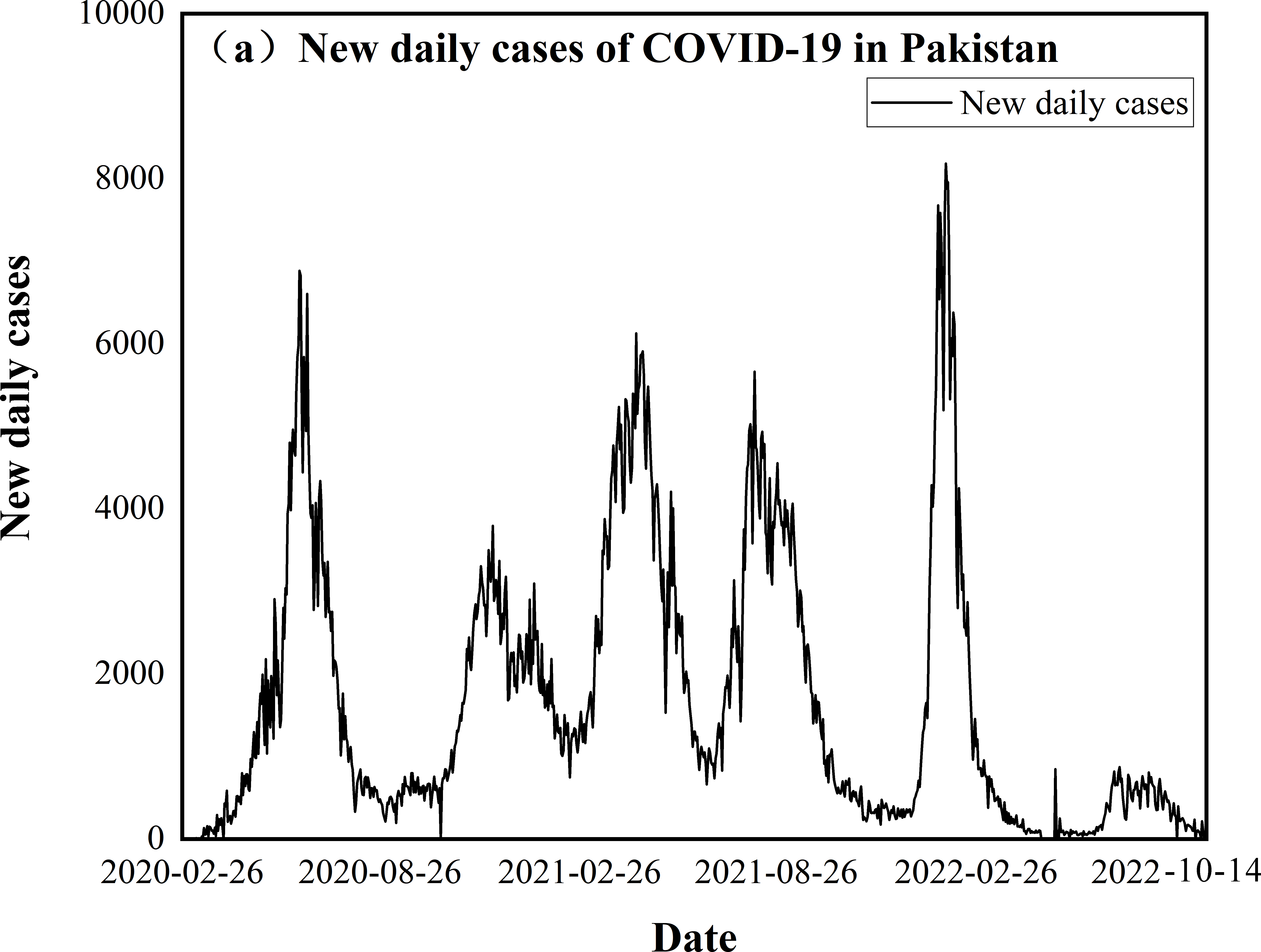

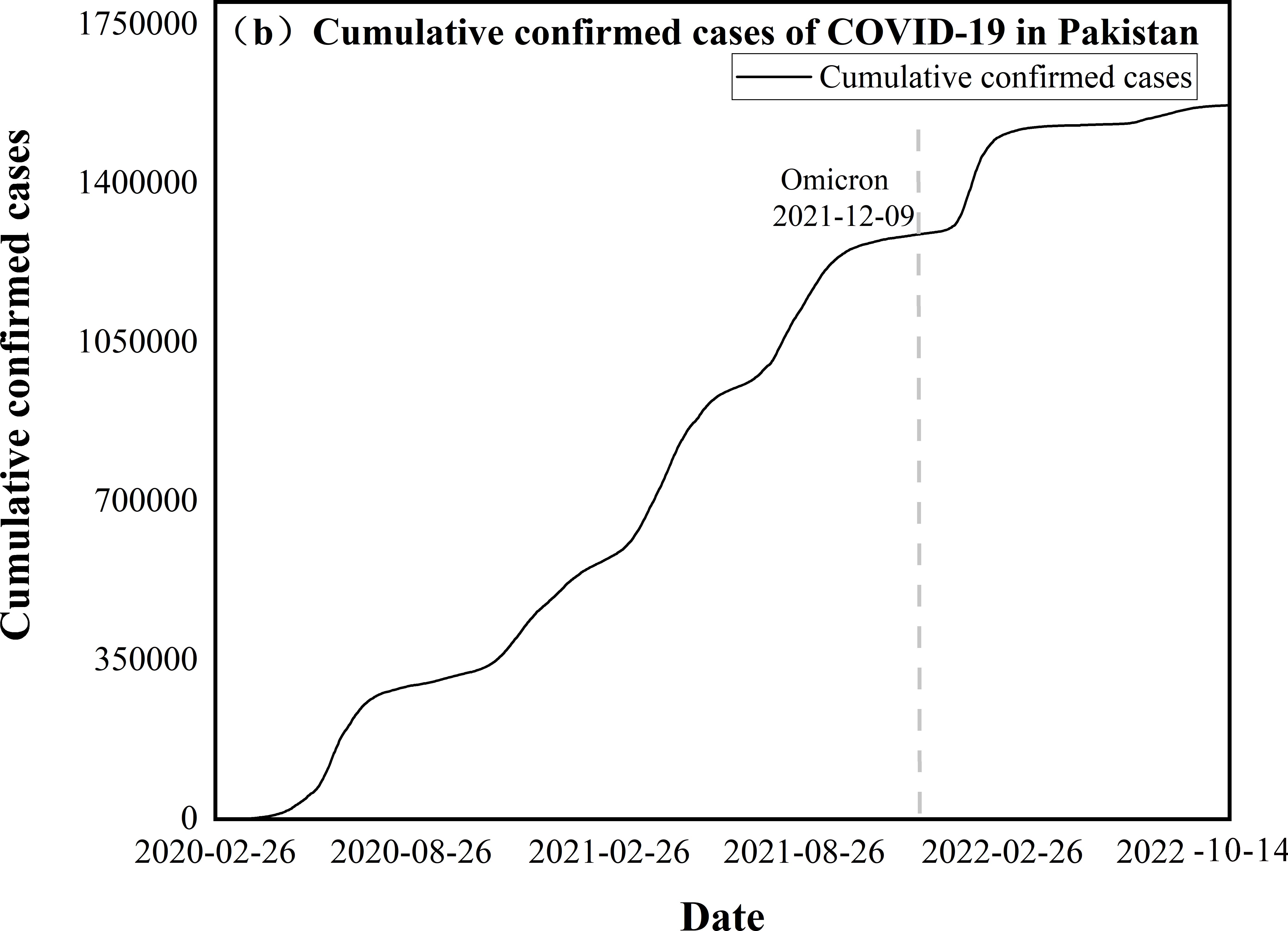


Figure S3: COVID-19 variations of Pakistan from February 26, 2020 to October 14, 2022, (a) for daily new cases and (b) for cumulated cases.
